# Application for Identifying the Origin and Predicting the Physiologically Active Ingredient Contents of *Gastrodia elata* Blume Using Visible–Near-Infrared Spectroscopy Combined with Machine Learning

**DOI:** 10.3390/foods12224061

**Published:** 2023-11-08

**Authors:** Jinfang Ma, Xue Zhou, Baiheng Xie, Caiyun Wang, Jiaze Chen, Yanliu Zhu, Hui Wang, Fahuan Ge, Furong Huang

**Affiliations:** 1 Department of Optoelectronic Engineering, Jinan University, Guangzhou 510632, China; 2 School of Pharmaceutical Sciences, Sun Yat-sen University, Guangzhou 510006, China; 3 Nansha Research Institute, Sun Yat-sen University, Guangzhou 511466, China; 4 Bijie Institute of Traditional Chinese Medicine, Bijie 551700, China

**Keywords:** *Gastrodia elata* Blume, visible–near-infrared spectroscopy, geographical origin, physiologically active ingredients, 1D-CNN

## Abstract

*Gastrodia elata (G. elata)* Blume is widely used as a health product with significant economic, medicinal, and ecological values. Due to variations in the geographical origin, soil pH, and content of organic matter, the levels of physiologically active ingredient contents in *G. elata* from different origins may vary. Therefore, rapid methods for predicting the geographical origin and the contents of these ingredients are important for the market. This paper proposes a visible–near-infrared (Vis-NIR) spectroscopy technology combined with machine learning. A variety of machine learning models were benchmarked against a one-dimensional convolutional neural network (1D-CNN) in terms of accuracy. In the origin identification models, the 1D-CNN demonstrated excellent performance, with the F1 score being 1.0000, correctly identifying the 11 origins. In the quantitative models, the 1D-CNN outperformed the other three algorithms. For the prediction set of eight physiologically active ingredients, namely, GA, HA, PE, PB, PC, PA, GA + HA, and total, the *RMSEP* values were 0.2881, 0.0871, 0.3387, 0.2485, 0.0761, 0.7027, 0.3664, and 1.2965, respectively. The $R_{p}^{2}$ values were 0.9278, 0.9321, 0.9433, 0.9094, 0.9454, 0.9282, 0.9173, and 0.9323, respectively. This study demonstrated that the 1D-CNN showed highly accurate non-linear descriptive capability. The proposed combinations of Vis-NIR spectroscopy with 1D-CNN models have significant potential in the quality evaluation of *G. elata*.

## 1. Introduction

In recent years, *Gastrodia elata (G. elata)* Blume has been used as a health product in some countries. Because it has some remarkable and reliable benefits, it has received very good attention. As one of the traditional food materials and a rare Chinese medicine, *G. elata* is widely used in cooking, healthcare products, and cosmetics in China [1]. *G. elata* was designated by the Chinese Health Commission as a pilot variety for the management of “substances that are both food and Chinese herbal medicines according to tradition” and represented a “medicine-food homology” in the legal sense [2,3]. Currently, most *G. elata* are artificially cultivated. The quality of *G. elata* may vary significantly between different geographical origins owing to the differences in the growing environment, climate, and soil [4]. In China, *G. elata* has been cultivated in many provinces, including Guizhou, Yunnan, Shaanxi, Hubei, and Henan. The World Health Organization (WHO) has indicated that origin certification is key to ensuring herbal medicines’ quality [5]. In pursuit of commercial interests, some inferior products were disguised as a *G. elata* protected by geographical indication (PGI). This not only affects the selling prices but also severely harms the interests of consumers. Therefore, the accurate identification of the geographical origin is essential to maintaining the good quality of the *G. elata* PGI.

Some research studies showed that there are many physiological active ingredients in *G. elata*. Due to the various physiologically active ingredients with significant nourishing and strengthening effects that contribute to the edible and medicinal values of *G. elata*, it is used in beverages and foods [6]. Most of these ingredients are phenolic compounds, such as gastrodin (GA), p-hydroxybenzyl alcohol (HA), parishin A (PA), parishin B (PB), parishin C (PC), and parishin E (PE) [7]. Therefore, accurate content determination of these physiologically active ingredients is of considerable importance.

Currently, high-performance liquid chromatography (HPLC) [8], elemental analysis [9], deoxyribonucleic acid barcoding [10], and other techniques are generally used to identify the origin of *G. elata*. Furthermore, HPLC [11,12], gas chromatography–mass spectrometry (GC-MS) [13], and liquid chromatography–mass spectrometry (LC-MS) [14] are used to determine the amounts of the physiologically active ingredients in *G. elata*. However, these analytical methods are time-consuming and complex, the chemical reagents used are subject to secondary contamination, and the required equipment is expensive. Consequently, they do not meet the current requirements for the evaluation of *G. elata* purchased at the geographical origin or on the open market. Therefore, the exploration of a low-cost, simple, and environmentally friendly evaluation method for *G. elata* is crucial.

Spectral technology is a relatively simple and fast evaluation method. Near-infrared spectroscopy (NIR) was applied to identify *G. elata* from four main geographical origins, with accuracy rates of 0.9700 and 0.9900 for the calibration and validation sets, respectively. When identifying samples from eight different cities, the accuracy rates for calibration and validation sets were 0.9800 and 0.9900, respectively. The prediction of the content of polyphenolic compounds yielded a determination coefficient of 0.9209 and a root-mean-square error of 0.3380 for the prediction set [15]. Excitation–emission matrix (EEM) fluorescence was successfully used to identify the geographical origin of *G. elata* from three regions with a 1.0000 accuracy on the training and test sets [16]. Fourier-transform infrared (FT-IR) spectroscopy was successfully used to identify *G. elata* from six geographical origins with a classification accuracy rate of 1.0000 [4]. NIR technology combined with TQ software was used to determine six effective components of *G. elata*. After the wavelength selection, the optimal quantitative models were obtained. The calibration set correlation coefficients for the quantification models of gastridin, p-hydroxybenzyl alcohol, parishin A, parishin B, parishin C, and parishin E were 0.9841, 0.9063, 0.9858, 0.9857, 0.9852, and 0.9506, respectively. The root-mean-square errors of calibration were 0.0427, 0.0170, 0.0749, 0.0269, and 0.0080, and the root-mean-square errors of prediction were 0.0837, 0.0116, 0.1380, 0.0699, 0.0145, and 0.1780, respectively [17]. In previous studies, there were no reports on the use of visible–near-infrared (Vis-NIR) spectroscopy for the identification of *G. elata* from different origins and the prediction of some physiologically active ingredients. Vis-NIR spectroscopy is valued for its simplicity, convenience, rapidity, and wide wavelength range for detection. It can be used to perform non-targeted spectroscopic chemical analysis for determining spectral fingerprints that can be applied to identify the samples [18]. The geographical origins of samples can be determined by assessing the spectral similarity, and any outliers can be identified [19]. Furthermore, Vis-NIR spectroscopy can be used to establish calibration models for the identification of components of interest, enabling the reliable and rapid determination of compounds. In recent years, Vis-NIR spectroscopy and machine learning algorithms have been closely tied in diverse fields, such as soil science [20], food science [21], and marine algae science [22]. However, the classical partial least squares regression (PLSR) and partial least squares discriminant analysis (PLS-DA) methods are not ideal for modeling complex non-linear systems owing to their inability to acquire non-linear features [23]. Comparatively, as a conventional machine learning algorithm, K-nearest neighbor (KNN) exhibits a high accuracy when it is used for classification and regression. However, it is insensitive to outliers and has issues, such as difficult feature band selection [24]. Meanwhile, support vector machine (SVM) and support vector regression (SVR), as non-linear algorithms, are effective in reducing the model complexity and prediction error for both classification and regression tasks. However, they require the manual selection of suitable features and kernel functions [25]. As a deep learning algorithm, the one-dimensional convolutional neural network (1D-CNN) can overcome the aforementioned issues.

So far, the application of convolutional neural networks (CNNs) has produced significant results in various fields. A 1D-CNN has a similar structure to conventional CNN; however, the former is more powerful at model representation than the latter. It can be trained effectively using limited data sets because it only requires simple pre-processing, exhibits good information extraction efficiency, and has low computational requirements. Therefore, optimal models can be obtained in real-life applications using a 1D-CNN [26,27,28]. Therefore, it was postulated that combining the excellent predictive performance of a 1D-CNN with detailed Vis-NIR analysis would allow for the simplification of complex tasks, such as the identification of the geographical origin and the prediction of the physiologically active ingredient contents in herbal medicines.

The aim of this study was to establish an effective and industrially referable method for the evaluation of *G. elata* to identify the origin of *G. elata* and predict its physiologically active ingredient contents. Specifically, our objectives were (1) to evaluate the potential of applying Vis-NIR spectroscopy for the identification of the geographical origin of *G. elata* and prediction of the bioactive contents, (2) to compare the prediction efficacy of models established using deep learning algorithms (1D-CNN) and conventional machine learning methods (PLS-DA/PLSR, KNN, and SVM/SVR) and identify the ideal modeling method, (3) to verify the feasibility of using a Vis-NIR discriminant model to identify the origin of *G. elata* through spectral characterization, and (4) to establish a calibration model for the bioactive components in *G. elata* using Vis-NIR analysis for predicting the contents of multiple components simultaneously.

## 2. Materials and Methods

### 2.1. Sample Collection and Pre-Treatment

**Figure 1:**
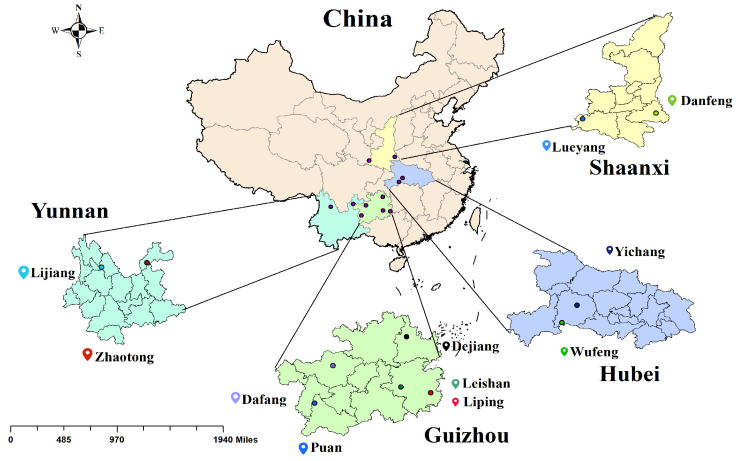
Geographical origin of the *G. elata* samples (highlighted with color).

The majority of the market’s *G. elata* comes from various geographical origins in China (Figure 1). The samples for this study were collected from these cultivation bases in December 2021 by the Bijie Institute of Traditional Chinese Medicine, Bijie, Guizhou Province. The information on the samples is shown in Table 1. The *G. elata* samples from different origins and batches were classified, cleaned, steamed for 30 min, and then dried in a 50 °C oven (Shanghai Yetuo Technology Co., Ltd., Shanghai, China). Subsequently, in order to have a homogeneous *G. elata* powder sample, each batch of dried *G. elata* samples was crushed and sieved. A total of 240 powder samples of *G. elata* were obtained and stored in a laboratory at room temperature (25 ± 1 °C) and a humidity of 45 ± 1%. These samples were analyzed via HPLC after collecting the Vis-NIR spectral data.

### 2.2. Acquisition of Vis-NIR Spectral Data

A Vis-NIR spectrometer (XDS Rapid Content, Foss NIR SystemsInc., Hillerød, Denmark) with silicon (400–1100 nm) and lead sulfide (1100–2500 nm) detectors was used to collect the spectra in the range of 400–2500 nm with a sampling interval of 2 nm. Vis-NIR spectra of samples were collected in the laboratory at a room temperature of 25 ± 1 °C and humidity of 45 ± 1%. The spectrum of each sample was measured three times, and the average spectrum was used for further analysis.

### 2.3. Determination of the Contents of Bioactive Components via HPLC

A total of 2.0 g of each *G. elata* sample was weighed and transferred into a 50 mL conical flask, and 25 mL of a 60% methanol solution was added. The mixture was weighed, and the extraction was performed via ultrasonication for 1 h. The mixture was then weighed, replenished, and centrifuged (Hunan Kaida Scientific Instrument Co., Ltd., Changsha, China). Thereafter, 5 mL of the supernatant was added to 5 mL of the 60% methanol solution and filtered through a 0.45 μm microporous membrane before analyzing via HPLC.

HPLC analysis was performed on an UltiMate 3000 HPLC system (Thermo Fisher Scientific, Waltham, MA, USA) with a Phenomenex Luna C18 (250 mm × 4.6 mm, 5 μm) column. The mobile phase consisted of acetonitrile (A) and 0.1% aqueous phosphate solution (B). The flow rate was 1.0 mL/min, and the gradient elution conditions were as follows: 0–5 min, 3.0% A; 5–15 min, 3.0–5.0% A; 15–22 min, 5.0% A; 22–25 min, 5.0–10.1% A; 25–35 min, 10.1–10.2% A; 35–45 min, 10.2–14.0% A; 45–52 min, 14.0% A; 52–55 min, 14.0–16.5% A; 55–63 min, 16.5–17.5% A; 63–65 min, 17.5–20.0% A; 65–70 min, 20.0% A. The column temperature was 35 °C, the injection volume was 4 μL, and the detection wavelength was 220 nm. For the validation of the HPLC method, refer to [29]. Compared with the method recorded in the Chinese Pharmacopoeia (2020) [30], this method allows for the simultaneous determination of multiple physiologically active ingredient contents of *G. elata*.

### 2.4. Chemometric Analysis

#### 2.4.1. Partial Least Squares Regression (PLSR)/Partial Least Squares Discriminant Analysis (PLS-DA)

PLSR is a multivariate statistical method commonly used in spectral analysis for regression with linear features. PLSR operates well when processing predictor variables with multicollinearity and considers both spectral and feature information. PLS-DA is an extension of the classical PLS algorithm and is also based on a linear classification technique [31,32]. In this study, the categorical variables of the calibration sample set were established first during the development of the PLS-DA discriminant model, followed by the PLS analysis of the categorical variables and the spectral data to establish their PLS model; the values of the categorical variable (ypredicted) of the testing set were calculated based on this PLS model. The classification variables of the eleven different sample origins were assigned as 1, 2, 3, 4, 5, 6, 7, 8, 9, 10, and 11. When 0.5 < ypredicted < 1.5, the samples were assigned to the first category. When 1.5 < ypredicted < 2.5, the samples were assigned to the second category.

#### 2.4.2. K-Nearest Neighbor (KNN)

KNN is a supervised-learning-based classification algorithm that can be used for classification and regression tasks [24]. The basic concept of the KNN algorithm [33] is to calculate the distance or similarity between the data of the sample to be classified and the known training samples and determine the K-nearest neighbors to the sample data to be classified according to the distance or similarity. Thereafter, the category of the sample data can be determined based on the categories of the neighbors. When all the K neighbors of the sample data belong to the same category, the sample is assigned to belong to that category. Therefore, new data Y and some training sample $X=\left(x_{1},x_{2},\ldots ,x_{n}\right)\;$ is usually calculated using the following Euclidean distance formula:

(1) $$d(x,y)=\sqrt{{\sum_{k=1}^{n}\left(x_{k}-y_{k}\right)}^{2}}$$

where $d\left(x,y\right)\;$ represents the Euclidean distance between Y and X. $x_{k}$ means the *k* feature attribute value of X training sample. $y_{k}$ means the *k* feature attribute value of the Y training sample.

#### 2.4.3. Support Vector Machine (SVM)/Support Vector Regression (SVR)

An SVM is a data mining method for classification and regression based on the structural risk minimization principle [25]. SVR is an application of SVM used to address regression problems and can model high-dimensional data well. Therefore, the radial basis function (RBF) kernel function was applied for SVM modeling in this research. The two important parameters of the RBF kernel function are the penalty parameter c and the kernel function parameter g. These factors are very important to the model, especially the complexity, approximation error, and measurement accuracy. In addition, their optimization is essential. Therefore, the grid search (GS) technique was used to select the optimal parameters for this study [34]. For this purpose, all the values of the c–g parameter pairs were tested, and the c–g pair with the highest accuracy was identified via cross-validation and used as the optimal parameters.

#### 2.4.4. One-Dimensional Convolutional Neural Network (1D-CNN)

**Figure 2:**
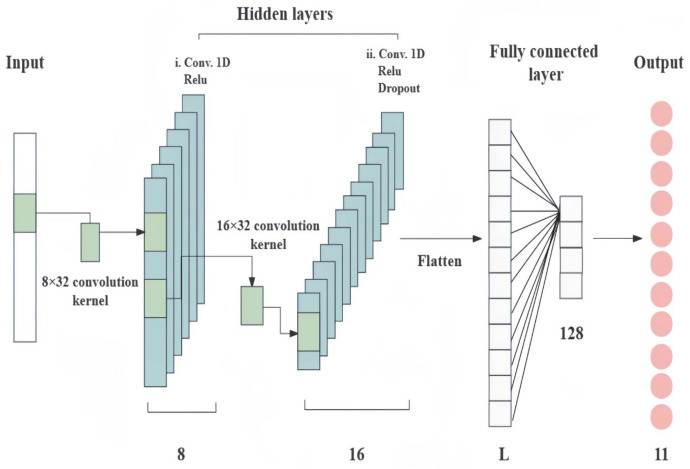
Components of a 1D-CNN.

The structure of a 1D-CNN is similar to that of a conventional CNN and consists of input, convolutional, grouping, pooling, fully connected, and dense layers (Figure 2) for feature extraction, learning, and providing numerical outputs for classification or regression tasks [35,36]. However, it is more powerful in model representation than a conventional CNN. The convolutional layer is composed of multiple convolutional kernels. Convolution with these kernels on the original data is regarded as extracting features that contain the characteristics represented by the convolutional kernels. The number of convolutional kernels determines the number of generated features. Different activation functions are used to show complex features. The dropout layer can temporarily discard a certain percentage of neural network units from each fully connected layer during the training process of a deep neural network. This means that different networks are trained for each batch, reducing the occurrence of overfitting. The fully connected layer maps the learned features to the sample label space. The flatten layer flattens the input data without affecting the batch size, serving as a transformation between the convolutional and fully connected layers.

The structure of the 1D-CNN models was built based on the TensorFlow framework, the structure and parameters of which are shown in Table 2. The default parameters in TensorFlow are not specified in the table. The input spectral data are presented as different origins. As Vis-NIR spectral data exhibits continuous changes in absorbance values with wavelength, when the convolutional kernel has a small size, the kernel may extract features on subintervals that are not near absorption peaks. Modeling with these features makes it difficult for the model to capture distinctive and discriminative spectral patterns, resulting in poor generalization performance. In addition, as shown in Figure 2, 8 kernels were used to capture low-level local features in the first convolutional layer. The second convolutional layer used 16 kernels to further combine and abstract these features, capturing higher-level features. This hierarchical feature extraction helped the model to better understand the structure of the data. Shallow networks have weaker modeling capabilities, and the combination of two convolutional layers could improve the model’s representational power. Therefore, a brief description of the layers is as follows:

(1) The Gaussian noise layer assisted in the regularization of the model by noising the pre-processed data with a Gaussian noise filter and was only valid during training. The value 1030 in the input shape of the Gaussian noise layer represented the feature dimension of each sample. In this study, the spectral data of each sample was obtained from the Vis-NIR spectra after spectral pre-processing and consisted of 1030 wavelength points. The Gaussian noise layer only added noise to the data without changing its shape, and thus, the output feature sequence remained 1030.

(2) In deep neural networks, the dimensionality of the input data is altered using the reshape layer. The reshape layer adapts the two-dimensional (2D) Vis-NIR spectroscopy data to three-dimensional (3D) data, with the third dimension having a fixed value of 1.

(3) There were two convolutional layers. The rectified linear unit (ReLU) function was regarded as the activation function of both convolutional layers. The convolutional layer 1 achieved convolution in 1D with 8 convolution kernels with a size of 32, with a stride of 1. Therefore, when data with 1030 features underwent convolution with a size of 32, the resulting number of features was (1030–32) + 1, which is 999 features. The third parameter, namely, 8, represented the number of convolutional kernels, each of which learned different features and generated an output sequence.

(4) The second convolutional layer further enhanced the model’s ability to learn feature representations and capture more feature information. Similarly, 16 convolutional kernels with a size of 32 were used in this layer. When data with 999 features underwent convolution with a size of 32, the resulting number of features was (999–32) + 1, which is 968 features. The third parameter, namely, 16, represented the number of convolutional kernels.

(5) The dropout layer generalized the model by randomly dropping out neurons to prevent overfitting, with the dropout rate of the input units represented by r.

(6) The flattening layer flattened the features extracted by the convolutional layers. During this process, it sequentially unfolded all the elements and transformed the output data shape to (None, 15,488).

(7) The dense layer, also known as the fully connected layer, further compressed the nodes in the network. It took an input dimension of 15488. This layer was configured with 128 neurons, each of which performed a weighted sum and processed the 15,488 input features, resulting in an output value.

(8) The output layer operated on the same principles as the previous fully connected layer. It was configured with 11 neurons, each of which performed a weighted sum and processed the 128 input features, resulting in an output value. It mapped the 128-dimensional features of the input to an 11-dimensional features space, where each neuron corresponded to a category, and the output value represents the probability of that category. Additionally, L1 and L2 losses were set in this layer to achieve weight regularization.

A 1D-CNN is more flexible in extracting key features and more expressive than conventional machine learning algorithms, enabling a better extraction of patterns and features. Furthermore, compared with conventional neural networks, 1D-CNN has fewer parameters and is easier to train.

### 2.5. Statistical Analysis

HPLC analysis requires known standards to identify the peaks representing GA, HA, PA, PB, PC, and PE. Based on the calibration curves, the content of each physiologically active ingredient can be calculated. The results of the PLS-DA, the principal component analysis (PCA), and a clustered heatmap were plotted to analyze the physiologically active ingredients.

For the model analysis, a total of 240 samples, as shown in Table 3, were divided in a 3:1 ratio between the training and testing sets using the sample set portioning based on the joint x–y distance (SPXY) algorithm. The SPXY method is a sample split method based on the Kennard–Stone (KS) method that can be used for the qualitative and quantitative analyses of spectra [37].

In this research, PLS, KNN, and SVM algorithm codes were produced using Python (ver.3.8.5, Python Software Foundation, Beaverton, OR, USA);scikit-learn package (ver. 1.0.2, https://github.com/scikit-learn/scikit-learn) (accessed on 22 October2023);keras (ver. 2.8.0, Google, Menlo Park, CA, USA); tensorflow (ver. 2.8.0, Google, Menlo Park, CA, USA). The 1D-CNN was performed using Python ver. 3.8.5 with the Keras library ver. 2.8.0 and the TensorFlow ver. 2.8.0 backend. All computations were carried out on a desktop PC with an Intel Xeon(R) Platinum 8124M 3.00 GHz processor, 64 GB RAM, and the NVIDIA GeForce RTX 3060.

Accuracy, precision, recall rate, and F1 score were used in this study; these are the evaluation indicators of the origin discrimination models. By comparing these indicators, the optimal Vis-NIR spectral discrimination model was selected to classify and validate the respective origins of the *G. elata* samples.

(2) $$\mathrm{Accuracy}=\frac{\mathrm{TP}+\mathrm{TN}}{\mathrm{TP}+\mathrm{TN}+\mathrm{FP}+\mathrm{FN}}$$

(3) $$\mathrm{Precision}=\frac{\mathrm{TP}}{\mathrm{TP}+\mathrm{FP}}$$

(4) $$\mathrm{Recall}=\frac{\mathrm{TP}}{\mathrm{TP}+\mathrm{FN}}$$

(5) $$F_{1}=\frac{2\mathrm{TP}}{2\mathrm{TP}+\mathrm{FP}+\mathrm{FN}}$$

where the numbers of true positive (*TP*), true negative (*TN*), false negative (*FN*), and false positive (*FP*) results were counted, respectively. The predictions also are shown in a confusion matrix for convenience [38].

The final optimal calibration model was chosen based on the minimum root-mean-square error of prediction (RMSEP), the highest coefficients of determination of the training set ($R_{v}^{2}$)and the prediction set ($R_{p}^{2}$), and the lowest mean relative errors for cross-validation (MRECV) and prediction (MREP). The regression model has a better fit when the coefficient of determination is closer to one. Moreover, numerically closer values of RMSECV and RMSEP suggest a better generalization ability of the model. When the results fulfilled these criteria, it could be concluded that the model was well suited for the prediction of physiologically active ingredient contents in *G. elata* from different origins.

## 3. Results

### 3.1. Statistical Analysis of Physiologically Active Ingredients Content Determined via the HPLC Method

The contents of the physiologically active ingredients (GA, HA, PA, PB, PC, and PE) in the *G. elata* samples from different origins were determined via HPLC. The sum of the contents of GA and HA [12], the sum of all component contents (total), and the coefficient of variation (CV) of each content were calculated (Table 4). Considering the CV values, the amounts and types of the *G. elata* samples from the eleven origins varied considerably. The GA content had the highest variation, with a CV of 61.06%, whereas the PB content had the lowest CV of 22.05%. The samples with a PGI indicating they were from DJ had the highest GA content of 4.7047 mg/g, whereas the samples with a PGI indicating that they were from YC had the lowest GA content of 1.0361 mg/g. The highest content of HA was 1.2674 mg/g in the samples with a PGI indicating that they were from LS, and the lowest HA content of 0.3898 mg/g was observed for samples from DAF. The highest content of PE was 5.8001 mg/g, which was detected for the samples from LP, and the lowest PE content of 1.9492 mg/g was observed for the samples from LY. The highest content of PB was 3.9674 mg/g in the samples with a PGI indicating that they were from DJ, and the lowest PB content was 2.0601 mg/g, which was detected for the samples from DF. The highest content of PC was 1.5170 mg/g in the samples with a PGI indicating that they were from DJ, and the lowest PC content was 0.4814 mg/g, which was detected in the samples from YC. The highest PA content was 10.3782 mg/g, which was determined in the samples with a PGI indicating that they were from DJ, and the lowest PA content was 2.6593 mg/g, which was detected in samples from ZT. The highest sum of the contents of GA and HA was 5.8882 mg/g and was detected in samples with a PGI indicating that they were from DJ, and the lowest value was 1.7200 mg/g obtained for samples from LP. The total contents ranged from a maximum of 24.4433 mg/g observed for samples with a PGI indicating that they were from DJ to a minimum of 12.0235 mg/g detected in samples from DAF. The reason for this phenomenon was that different regions lead to differences in soil pH, organic matter content, and microbial populations [39]. As the contents of the physiologically active ingredients in the *G. elata* are correlated with these influencing factors, the levels of physiologically active ingredients in the *G. elata* from different regions varied.

The results of the comparison show that the *G. elata* samples with a PGI indicating that they were from DJ had the highest physiologically active ingredients content, which may be attributed to the fact that this region has a humid climate, sufficient sunlight, average temperatures of 13–17 °C throughout the year, and frost-free weather up to 295 days a year [40]. The *G. elata* samples with a PGI indicating that they were from LS had lower physiologically active ingredient contents than the samples from PUA without a PGI. The total contents in the *G. elata* samples collected from DAF and ZT with PGIs were lower than those in samples collected from PUA, LP, and WF without PGIs. The season of collection and the grade of the collected *G. elata* may have both contributed to the poor quality of samples collected from DAF and ZT. This finding indicates that sourcing *G. elata* from a region with a PGI does not ensure high physiologically active ingredient contents. Furthermore, the quality of *G. elata* should be determined based on a combination of the origin and physiologically active ingredient contents of the sample.

**Figure 3:**
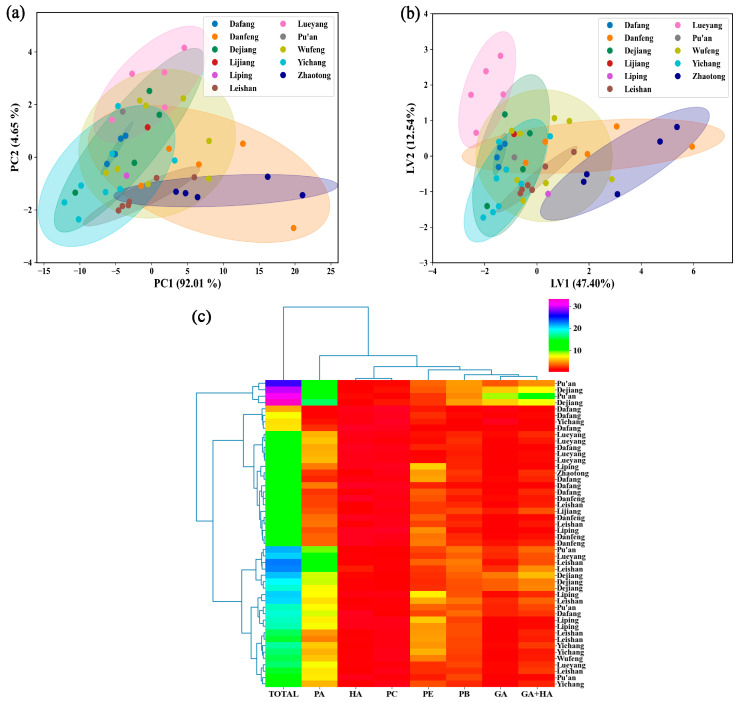
Multi-indicator analysis of the origin of *G. elata*: (**a**) PCA, (**b**) PLS-DA, and (**c**) clustered heatmap. GA: gastrodin; HA: p-hydroxybenzyl alcohol; PE: parishin E; PB: parishin B; PC: parishin C; PA: parishin A; GA + HA: the sum of gastrodin and p-hydroxybenzyl alcohol; total: the sum of gastrodin, p-hydroxybenzyl alcohol, parishin E, parishin B, parishin C, and parishin A.

For a more intuitive study, we combined the analysis of the physiologically active ingredient contents in *G. elata* with chemometrics to identify the clusters of origins. The 2D PCA score plot for the origin classification (Figure 3a) showed that the sum of PC1 and PC2 accounted for 96.65% of the explained total variance (PC1 = 92.01%, PC2 = 4.65%) and could explain most of the variability. However, no significant clustering of the samples from the eleven origins was observed, and the data points were dispersed and had overlapping distributions. Based on the results of the PLS-DA classification (Figure 3b), the samples from several origins showed indications of classification, but ultimately, they could not be completely separated owing to the extensive overlaps. In addition, to examine whether certain components were indicative of certain origins, a clustered heatmap analysis of the physiologically active ingredients content (*x*-axis) and sample origin (*y*-axis) was performed (Figure 3c). The results showed that the samples from the same origin were not distinguished, indicating that the physiologically active ingredient contents varied in samples from the same origin. This may have been because the *G. elata* samples had different quality grades [41]. The results of the aforementioned analysis indicate that it is not currently possible to identify the origin of *G. elata* by solely considering the physiologically active ingredient contents. Therefore, efficient methods should be developed to rapidly and accurately identify the origin of *G. elata*.

### 3.2. Analysis Based on the Vis-NIR Method

#### 3.2.1. Spectral Analysis of the *G. elata* Samples

**Figure 4:**
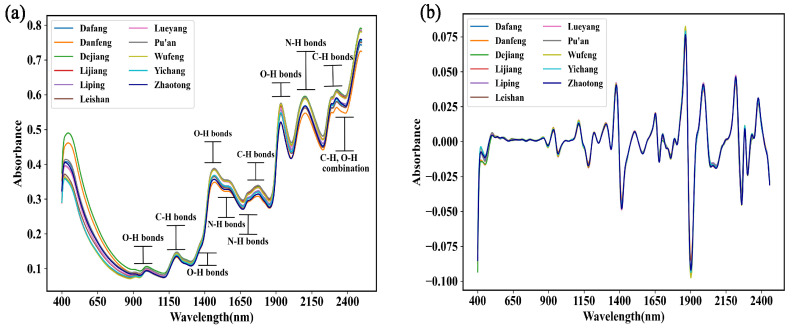
Vis-NIR characterization of *G. elata*: (**a**) original averaged spectra of samples from each origin and (**b**) averaged spectra of samples from each origin after second-order derivative (SD) processing.

Figure 4a shows that there were some average raw spectra of the *G. elata* samples that represented different origins, and there were eight distinct peaks and seven valleys. These average spectra that represent different origins were consistent in trends and overlapped tightly; however, subtle differences were observed in the absorption intensities, particularly in the Vis detection range (400–780 nm). The absorption peaks at approximately 980 nm referred to the second harmonic generation (SHG) of O–H in the phenolic acids and water. The absorption peaks at ~1180 nm referred to the SHG of C–H in the phenolic acids and polysaccharides in *G. elata*. The absorption peaks in the range of 1420–1440 nm referred to the first harmonic generation (FHG) of O–H in the phenolic acids and polysaccharides in *G. elata*, as well as in water. The absorption peaks in the range of 1500–1600 nm referred to the FHG of C–H in the phenolic acid and polysaccharides in *G. elata* and the SHG of N–H in amino acids. The absorption peaks at 1940 nm referred to the sum frequency generation (SFG) of O–H in water, and those at 2150 and 2350 nm referred to the SFG of N–H in amino acids and C–H in phenolic acids in *G. elata*, respectively.

To reduce noise, the Vis-NIR spectra were pre-treated with a second-order derivative (SD) in this study, which significantly changed the shapes of the spectra (Figure 4b). The intensities of certain peaks, such as those at 1400 and 1900 nm, were enhanced. However, distinguishing the origin of *G. elata* using the averaged spectrum remains difficult.

#### 3.2.2. Visual Analysis of Spectral Characteristics

**Figure 5:**
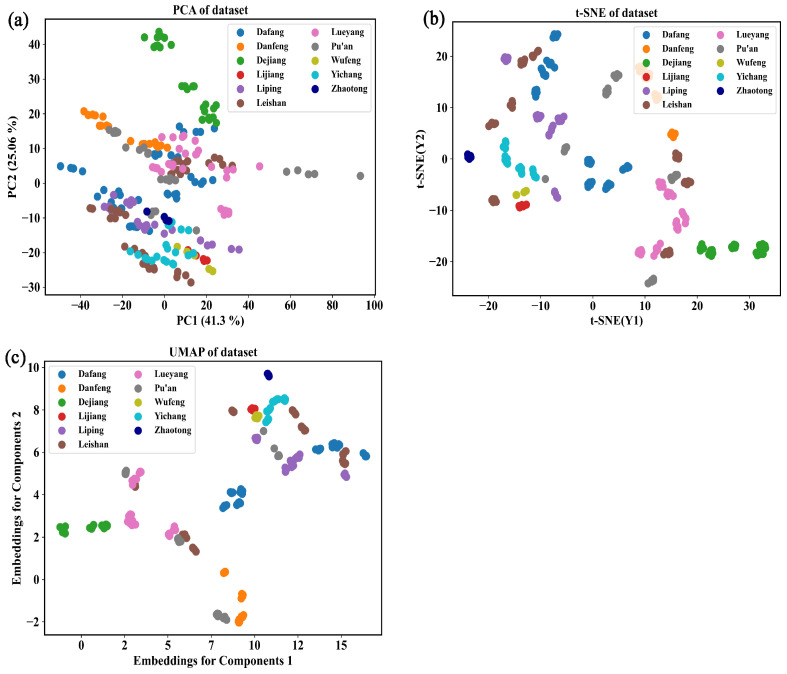
Visualization of the spectral characteristics of *G. elata*: (**a**) PCA visualization, (**b**) t-SNE visualization, and (**c**) UMAP visualization.

To understand the similarities and differences in the Vis-NIR spectral datasets of *G. elata* samples obtained from 11 different origins more intuitively and fully, three methods were used to map the Vis-NIR spectral datasets pre-processed with SD to a 2D space. As shown in Figure 5a–c, where each point represents an individual sample, visualization analysis was performed on the 11 datasets.

The 2D score plot obtained from PCA can cluster samples with similar spectral characteristics together [31,32]. The results show that the sum of PC1 and PC2 for origin classification accounted for 66.3% of the explained total variance (PC1 = 41.2%, PC2 = 25.1%). Figure 5a presents that except for the samples from DJ, the samples from other regions overlapped to a large extent, indicating poor separation. The t-distributed stochastic neighbor embedding (t-SNE) visualization results (Figure 5b) show that the samples from DF, DJ, LJ, and WF formed distinct clusters, while samples from other regions exhibited significant overlap, indicating poor classification. The difference between the uniform manifold approximation and projection (UMAP) and t-SNE was minimal (Figure 5c). Although PCA is an effective method for extracting data information, it was not able to visualize a large amount of information. Comparatively, as a non-linear dimensionality reduction method, the t-SNE visualization method visualized the data significantly better than PCA [42]. The UMAP approach and computation were largely similar to t-SNE [43]. Compared with previous research, the results of PCA are consistent with those of the literature [15], but the results of t-SNE and UMAP are superior to the previous research. The visualization results validate the fact that the identification of *G. elata* origin cannot be accomplished by solely considering the clustering of spectra. At the same time, it was shown that *G. elata* from different origins had similar chemical compositions. Therefore, the combined application of Vis-NIR spectroscopy and chemometrics is required for further analysis.

#### 3.2.3. Identification of the Origin of *G. elata* Based on the Vis-NIR Data

The Vis-NIR spectral data were analyzed using 1D-CNN and other learning algorithms (PLS-DA, KNN, and SVM), employing the eleven origins as labels. The results are shown in Table 5. To enhance the stability of the model and avoid overfitting, the original spectral data sets were expanded by adding random offsets and applying multiplication and slope effects. The random offset of the spectral data was set to 0.1 times the mean value, and the slope was offset by 0.05 times, that is, the slope was randomly adjusted between 0.95 and 1.05 to augment the spectrum. The data augmentation method was used to obtain a total of 1440 spectra for the training set. The training set was then used to train the neural network and to avoid the risk of developing models with poor generalization ability. The same expanded training and validation sets were also applied to other chemometric methods to compare the advantages and disadvantages of models developed using 1D-CNN and other chemometric methods before and after the data augmentation.

When analyzing the models developed with unexpanded data, it was found that the results of the raw spectra performed the worst. A comparison of the other spectral pre-processing methods for the models developed with unexpanded data showed that the classification outcomes of all four models were improved after the spectral data were pre-processed via a combination of SD and normalization methods. Specifically, the training set accuracy (Acc_train) and the testing set accuracy (Acc_test) of the PLS-DA model improved by 6.07% and 4.24%, respectively, after SD processing. Meanwhile, the precision improved by 4.37%, and the recall rate and the F1 score improved by 2.98% and 4.57%, respectively. Although the Acc_train of the KNN model was reduced, the Acc_test of the KNN model improved from 0.5167 to 0.9667, the precision improved from 0.5060 to 0.9542, the recall rate improved from 0.5167 to 0.9667, and the F1 score improved from 0.5062 to 0.9583. Additionally, the model performance of SVM improved considerably after the SD processing of the spectra. Acc_train improved from 0.8278 to 0.9611, Acc_test improved from 0.6500 to 0.9833, the precision improved from 0.7211 to 0.9847, the recall rate improved from 0.6500 to 0.9833, and the F1 score improved from 0.6579 to 0.9828. Similarly, the 1D-CNN model performance improved after SD processing, obtaining a value of 1.0000 for each of Acc_train, Acc_test, precision, recall rate, and F1 score when using processed data. As a result of a comprehensive comparison of the models established with unexpended data, the optimal model of *G. elata* origin discrimination could be established by pre-processing the Vis-NIR spectra using SD and normalization combined with the application of 1D-CNN.

Analyzing the models established based on expanded data, it was found that the results of the raw spectra performed the worst. A comparison of the two spectral pre-processing methods for the models established based on expanded data suggested that the classification results of all four models were improved after the spectral data was pre-processed with both SD and normalization. Specifically, after pre-processing, for the PLS-DA model, Acc_train and Acc_test improved by 1.00% and 7.27%, respectively; the precision improved by 6.41%, and the recall rate and F1 score improved by 7.27% and 7.29%, respectively. In the case of the KNN model, Acc_test improved from 0.7167 to 0.9833, precision improved from 0.7409 to 0.9861, the recall rate improved from 0.7167 to 0.9833, and the F1 score improved from 0.7121 to 0.9829. The SVM model exhibited a significant improvement in parameters, with Acc_train improving from 0.8625 to 1.0000, Acc_test improving from 0.8833 to 0.9833, precision improving from 0.9012 to 0.9917, the recall rate improving from 0.8833 to 0.9833, and the F1 score improving from 0.8718 to 0.9849. Finally, the performance of the 1D-CNN model was improved, with the values of Acc_train, Acc_test, precision, recall rate, and F1 score equal to 1.0000. A comprehensive comparison of all the models developed with expanded data revealed that the optimal model for discriminating the origin of *G. elata* was the combined application of Vis-NIR spectra pre-processed using both SD and normalization and the 1D-CNN method.

The performances of the models established before and after data augmentation were compared after the spectral data were pre-treated via normalization. The identification results of the PLS-DA, KNN, SVM, and 1D-CNN models were improved after the augmentation of the Vis-NIR spectral data. The 1D-CNN model exhibited the highest performance, with an Acc_train of 1.0000, an Acc_test of 0.9833, a precision of 0.9847, a recall rate of 0.9833, and an F1 score of 0.9833. Thereafter, the performances of the models developed before and after data augmentation after the spectral data were pre-processed using both SD and normalization were compared. The classification results of the PLS-DA, KNN, SVM, and 1D-CNN models were also improved after the Vis-NIR spectral data augmentation. The 1D-CNN model showed the optimal results, with the values of the Acc_train, Acc_test, precision, recall rate, and F1 score equal to 1.0000. These results of 1D-CNN models also showed the presence of more inherent nonlinear correlations between spectral data and the original labels. Therefore, data augmentation is a viable Vis-NIR spectral data set augmentation technology. It improves the robustness of the 1D-CNN model.

**Figure 6:**
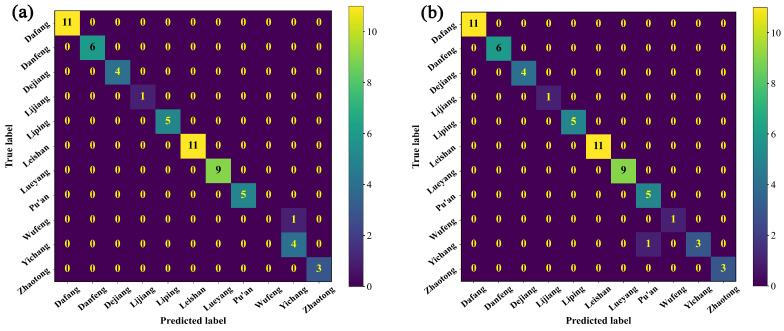

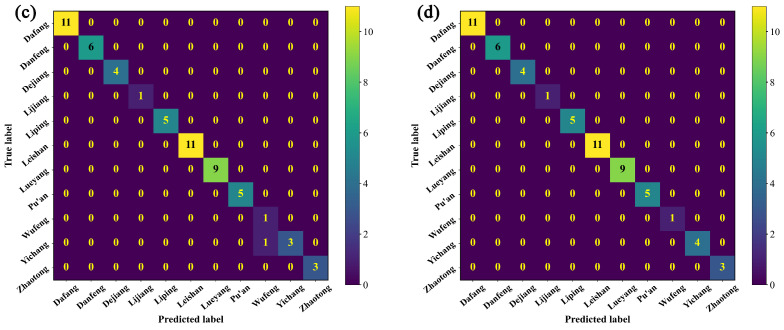
Confusion matrices: (**a**) PLS-DA, (**b**) KNN, (**c**) SVM, and (**d**) 1D-CNN.

In conclusion, the optimal spectral pre-processing method combined pre-processing with SD and normalization. Furthermore, the robustness of the model could be improved using data augmentation, and the optimal modeling algorithm was the 1D-CNN. To further verify the performances and effectiveness of the classification models in this study, the optimal model of each of the four algorithms was selected for plotting their confusion matrices, aiming to apply different discriminant models to each sample to obtain further details (Figure 6). The confusion matrices indicate that one sample from WF was misclassified as being from YC in the PLS-DA model, one sample from YC was misclassified as being from PA in the KNN model, and one sample from YC was misclassified as being from WF in the SVM model. Notably, all samples were correctly classified when using the 1D-CNN model. These results indicate that the PLS-DA, SVM, and KNN algorithms confused the data of the *G. elata* samples collected from YC, WF, and PA during classification, which may have been because of the characteristic spectral bands that suggest the differences in the origin and the bioactive component contents of *G. elata*. The 1D-CNN algorithm effectively addressed these issues. Therefore, the optimal model was established by pre-processing the Vis-NIR spectra using both SD and normalization, expanding the data, and modeling with the 1D-CNN algorithm. The results confirmed that the 1D-CNN model had strong automatic learning characteristics and was better suited for the origin identification of *G. elata* than the other models considered in this research, providing a rapid method for distinguishing samples of different origins with PGI.

**Figure 7:**
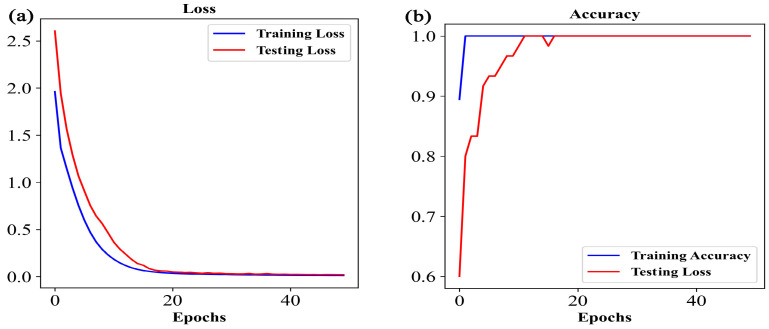
Trend chart of loss and accuracy in 1D-CNN training process: (**a**) Loss curves and (**b**) accuracy curves.

The loss and accuracy curves of the training set and testing set can diagnose any issues that could be causing underfit or overfit models during the learning process [44]. If the model is overfitting, the loss curve will gradually decrease on the training set, but it may stabilize or start to increase on the testing set. The accuracy of the model on the training set may approach 100%, while it may decrease or stabilize on the testing set. If the model is underfitting, both the loss curve on the training set and the testing set may fail to reach a low level, and the difference between them may be small. The accuracy of the model on both the training set and the testing set may be low, with a small difference between them. As a result, the 1D-CNN model was trained with an initial learning rate of 0.01, 50 iterations (epoch = 50), and a batch size of 32 (batch_size = 32) in this study. From Figure 7a, it can be observed that as the number of training iterations increased, the loss function of the 1D-CNN model for both the training set and testing set gradually decreased, indicating that the model was finding spectral features related to the origin of *G. elata*. When the loss function was very low, the loss curve decreased significantly and became flat as it approached zero. At this point, the 1D-CNN model became more stable, and the losses of the training set and testing set both converged, with a small difference between them, indicating successful fitting. Figure 7b shows that the accuracy curve of the 1D-CNN model on both the training set and testing set approached 1 (or 100%), indicating optimal model performance.

In terms of origin identification, the relevant literature has identified *G. elata* from up to eight different regions [15]. In this study, successful identification of *G. elata* from 11 different regions was achieved. From the perspective of the algorithm performance, the identification accuracy of the 1D-CNN model was comparable to the accuracy reported in the literature for three or six different regions of *G. elata* [4,16].

In addition, the evaluation indicators of the 1D-CNN model in this study were consistent with those in the relevant literature, and the F1 score was 1 [45]. The main reason for this was that 1D-CNN has advantages, such as local feature extraction, parameter sharing, multi-level abstraction, and non-linear activation functions, which enable it to capture the correlations between data more effectively. In this study, although the *G. elata* samples were sourced from different regions, they were harvested during the same period and under the same cultivation techniques. With fewer confounding factors in the experiment, the 1D-CNN model was able to capture the correlations between the data more easily, resulting in an F1 score value of 1.0000. Therefore, future research should collect more *G. elata* samples from different regions that are harvested at different times, in order to enhance the reliability and applicability of the 1D-CNN model.

#### 3.2.4. Prediction of Physiologically Active Ingredient Contents in *G. elata* Based on the Vis-NIR Method

Considering the intrinsic association between the origins of *G. elata* samples and their contents of physiologically active ingredients, it was investigated whether the Vis-NIR technique could be used to predict the contents of ingredients in *G. elata*. In this study, the SPXY method was applied to divide the training and testing sets (Table 3). The X variable was the Vis-NIR spectra after pre-processing using both SD and normalization and the Y variable was the content of each component in the samples determined via HPLC. The X variable was set to 180 samples × 1050 variables before the data augmentation and 1440 samples × 1050 variables after the data augmentation.

**Figure 8:**
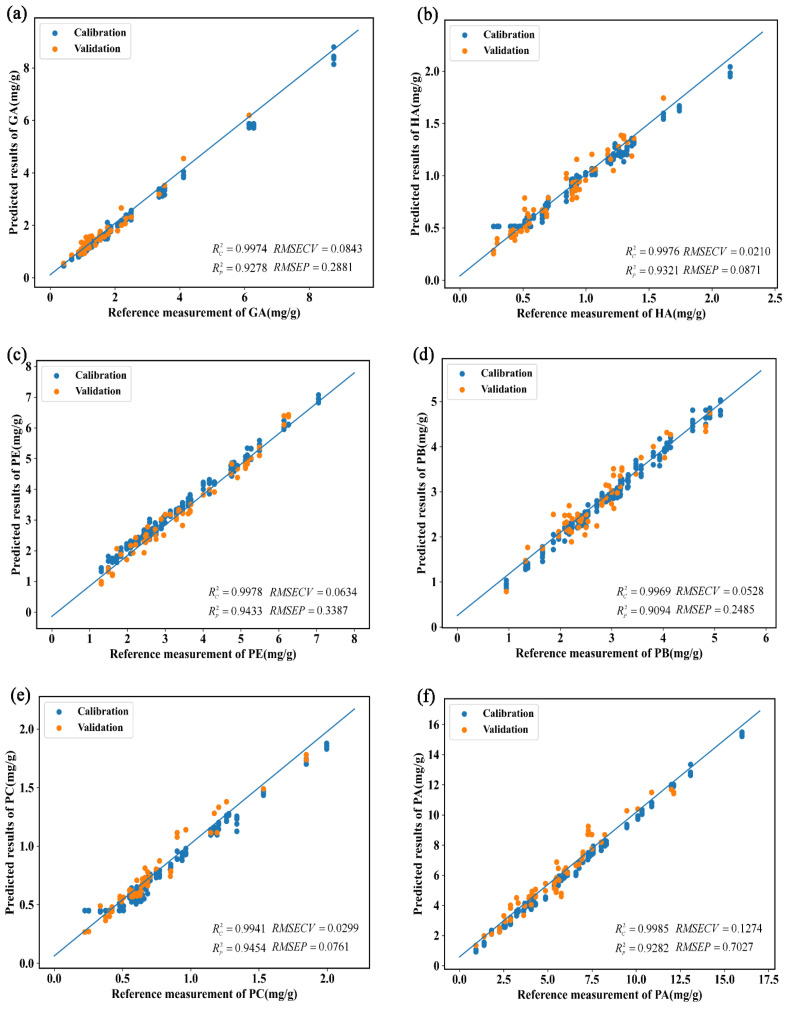

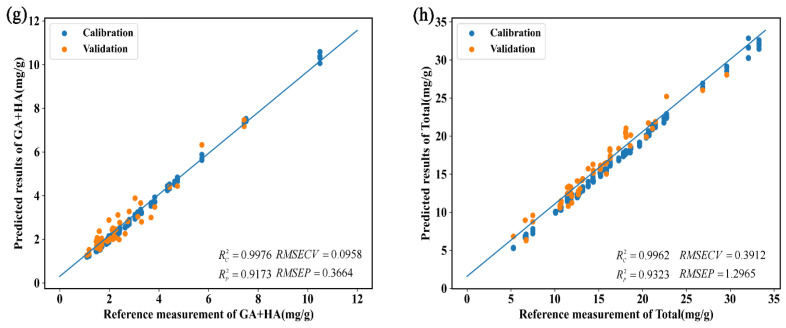
Graphical relationships between reference values and predicted values of the optimal calibration models for (**a**) gastrodin (GA); (**b**) p-hydroxybenzyl alcohol (HA); (**c**) parishin E (PE); (**d**) parishin B (PB); (**e**) parishin C (PC); (**f**) parishin A (PA); (**g**) the sum of gastrodin and p-hydroxybenzyl alcohol (GA + HA); and (**h**) the sum of gastrodin, p-hydroxybenzyl alcohol, parishin E, parishin B, parishin C, and parishin A (total).

The parameters of the model for determining the physiologically active ingredients content of *G. elata* based on the Vis-NIR full-wavelength spectra are listed in Table 6. When the data augmentation was not performed, the GA content was most effectively predicted by the SVR and 1D-CNN models, as both models had high $R_{v}^{2}$ (higher than 0.9800) and $R_{p}^{2}$ values (higher than 0.8800). After the data augmentation, the predictive performance of PLSR and SVR did not improve significantly. However, the performances of the KNN and 1D-CNN models improved, yielding $R_{v}^{2}$ and $R_{p}^{2}$ values higher than 0.9900 and 0.9200, respectively. This indicates that the KNN and 1D-CNN models were more precise in predicting GA content after the data augmentation than the other models. Comparing the performance parameters of the models, the optimal method was determined to be the combined application of the 1D-CNN algorithm and the expanded Vis-NIR spectral data pre-processed using SD (Figure 8a). The optimal model had the highest $R_{v}^{2}$ and $R_{p}^{2}$ values (0.9974 and 0.9278, respectively) and lower RMSECV, RMSEP, MRECV, and MREP values (0.0843, 0.2881, 0.0328, and 0.1396, respectively) than the other models, suggesting that it was the optimal model for predicting the GA content.

Among the values of the models used to predict the HA content, the R2 values of the PLSR, KNN, SVR, and 1D-CNN models were higher than 0.9100 when the training data was used without data augmentation, and the values of the KNN, SVR, and 1D-CNN models were particularly high (all values higher than 0.8700). After the data augmentation, the *R*^2^ values of the PLSR model improved significantly, and the $R_{v}^{2}$ values of the KNN model decreased. However, the $R_{v}^{2}$ values of the SVR and 1D-CNN models improved and were higher than 0.9700. Meanwhile, their $R_{p}^{2}$ values were higher than 0.8900, indicating that the SVR and 1D-CNN models were more accurate in predicting the HA content than the other models. Comparing the performance parameters of the models, the optimal model involved the combined application of the 1D-CNN algorithm and the expanded Vis-NIR spectral data pre-processed using SD (Figure 8b). This optimal model had the highest $R_{v}^{2}$ and $R_{p}^{2}$ values (0.9976 and 0.9321, respectively) and lower RMSECV, RMSEP, MRECV, and MREP values (0.0210, 0.0871, 0.0201, and 0.0884, respectively) compared with the other models, suggesting it was the best model for predicting the HA content.

Among the models used for predicting the PE content, only the 1D-CNN model had high $R_{v}^{2}$ (0.9971) and $R_{p}^{2}$ (>0.8963) values when no data augmentation was performed. After the data augmentation, the performance of the PLSR model was not improved significantly, whereas the KNN, SVR, and 1D-CNN models exhibited $R_{v}^{2}$ and $R_{p}^{2}$ values higher than 0.9300 and 0.9200, respectively. This indicates that the KNN, SVR, and 1D-CNN models were more accurate in predicting the PE content than PLSR. Comparing the performance parameters of the models, the optimal model involved the combined application of the 1D-CNN algorithm and the expanded Vis-NIR spectral data pre-processed using SD (Figure 8c). The optimal model had the highest $R_{v}^{2}$ and $R_{p}^{2}$ values (0.9978 and 0.9433, respectively) and lower RMSECV, RMSEP, MRECV, and MREP values (0.0634, 0.3387, 0.0164, and 0.0839, respectively) compared with other models, suggesting it was the best model for predicting the PE content.

Among the models used for predicting the PB content, the KNN, SVR, and 1D-CNN models had high values of $R_{v}^{2}$ (all greater than 0.9100) and $R_{p}^{2}$ (all greater than 0.8300) when no data augmentation was performed. After the data augmentation, the performances of the PLSR and KNN models were worse than before the augmentation, while the SVR and 1D-CNN models both had $R_{v}^{2}$ values greater than 0.9900 and $R_{p}^{2}$ values greater than 0.9000. This indicates that the SVR and 1D-CNN models had higher accuracy in predicting the PB content after the data augmentation. By comparing the performance parameters of the models, it was concluded that the optimal model comprised the combined use of the 1D-CNN algorithm and the expanded Vis-NIR spectra data pre-processed with SD (Figure 8d). The optimal model had the highest $R_{v}^{2}$ and test R^2^ values (0.9969 and 0.9094, respectively) and lower RMSECV, RMSEP, MRECV, and MREP values (0.0528, 0.2485, 0.0151, and 0.0788, respectively) than the other models, suggesting it was the best model for predicting the PB content.

Among the models used for predicting the PC content, the KNN, SVR, and 1D-CNN models had $R_{v}^{2}$ and $R_{p}^{2}$ values higher than 0.9500 and 0.8500, respectively, when no data augmentation was performed. After data augmentation, the property of the PLSR model was decreased, and the $R_{v}^{2}$ value of the KNN model was reduced. However, the $R_{v}^{2}$ values of the SVR and 1D-CNN models were higher than 0.9600, and the $R_{p}^{2}$ values were higher than 0.8600, indicating that the SVR and 1D-CNN models were more accurate in predicting the PC content after the data augmentation than the other models. Comparing the performance parameters of the models, the optimal model involved the combined application of the 1D-CNN algorithm and the expanded Vis-NIR spectral data pre-processed using SD (Figure 8e). The optimal model had the highest $R_{v}^{2}$ and $R_{p}^{2}$ values (0.9941 and 0.9454, respectively), as well as lower RMSECV, RMSEP, MRECV, and MREP values (0.0299, 0.0761, 0.0335, and 0.0887, respectively), compared with the other models, suggesting that it was the best model for predicting the PC content.

Among the models used for predicting the PA content, the $R_{v}^{2}$ and $R_{p}^{2}$ values of the KNN, SVR, and 1D-CNN models were higher than 0.9300 and 0.8400, respectively, when no data augmentation was performed. After the data augmentation, the performance of the PLSR model was not significantly improved, and the $R_{v}^{2}$ value of the KNN model decreased. However, the $R_{v}^{2}$ values of the SVR and 1D-CNN models increased and were higher than 0.9900, whereas the $R_{p}^{2}$ values were higher than 0.9000. This indicates that the SVR and 1D-CNN models were more accurate than the other models in predicting the PA content after the data augmentation. Comparing the performance parameters of the models, the optimal model involved the combined application of the 1D-CNN algorithm and the expanded Vis-NIR spectral data pre-processed using SD (Figure 8f). This optimal model possessed the highest $R_{v}^{2}$ and $R_{p}^{2}$ values (0.9985 and 0.9282, respectively) and lower RMSECV, RMSEP, MRECV, and MREP values (0.1274, 0.7027, and 0.0027, respectively) than the other models, suggesting it was the best model for predicting the PA content.

Among the models used to predict the GA + HA content, the KNN, SVR, and 1D-CNN models all had $R_{v}^{2}$ values greater than 0.9100 and $R_{p}^{2}$ values greater than 0.8900 when no data augmentation was performed. After the data augmentation, the performance of all three models was improved; however, as with the PLSR model, none of the improvements were significant. By comparing the performance parameters of the models, it was concluded that the optimal model comprised the combined use of the 1D-CNN algorithm and the expanded Vis-NIR spectra data pre-processed with SD (Figure 8g). This model had the highest values of $R_{v}^{2}$ and $R_{p}^{2}$ (0.9976 and 0.9173, respectively) and lower RMSECV, RMSEP, MRECV, and MREP values (0.0958, 0.3664, and 0.3664, respectively) compared with the other models, suggesting it was the best model for predicting GA + HA content.

Among the models used for predicting the total content of physiologically active ingredients, the KNN, SVR, and 1D-CNN models exhibited $R_{v}^{2}$ and $R_{p}^{2}$ values higher than 0.9300 and 0.8400, respectively, when no data augmentation was performed. After the data augmentation, the performance of all three models improved, with $R_{p}^{2}$ and $R_{p}^{2}$ values higher than 0.9700 and 0.9200, respectively. This suggests that these three models were more accurate than the other models in predicting the total content of physiologically active ingredients after data augmentation. However, the performance of the PLSR model did not improve after the data augmentation. Comparing the performance parameters of the models, the optimal model involved the combined application of the 1D-CNN algorithm and the expanded Vis-NIR spectral data pre-processed using SD (Figure 8h). This optimal model had the highest $R_{v}^{2}$ and $R_{p}^{2}$ values (0.9962 and 0.9323, respectively) and lower RMSECV, RMSEP, MRECV, and MREP values (0.3912, 1.2965, and 0.065, respectively) compared with the other models, suggesting that it was the best model for predicting the total content of physiologically active ingredients.

In summary, based on the holistic nature of Vis-NIR spectra, combined with the effectiveness in extracting the feature structure and strong modeling ability of 1D-CNN, multiple physiologically active ingredient contents in *G. elata* from different origins can be rapidly and simultaneously predicted. (1) By comparing different algorithms, it was concluded that the model built using the SD-pre-processed Vis-NIR spectra, data augmentation, and 1D-CNN algorithm had the highest predictive ability. This further demonstrated that the 1D-CNN model was capable of describing non-linear relationships better than the other models. (2) Without the data augmentation, the optimal quantitative modeling algorithms for predicting the contents of PB and GA + HA were SVR and 1D-CNN, whereas the optimal quantitative modeling algorithm for predicting the contents of other physiologically active ingredients was 1D-CNN. (3) After the data augmentation, the optimal quantitative modeling algorithm for predicting the contents of all the physiologically active ingredients was 1D-CNN, which demonstrates that data augmentation can improve the generalization ability of the 1D-CNN model. The relevant literature predicted the content of up to six physiologically active ingredients from *G. elata* [17]. In this study, successful prediction of the content of up to eight physiologically active ingredients was achieved. Moreover, the 1D-CNN model outperformed the methods presented in the literature regarding predicting ingredients.

## 4. Discussion

The main factors that affect the quality of *G. elata* are its origin and physiologically active ingredients. There are marked differences in the content of physiologically active ingredients in *G. elata* from 11 geographical origins, which confirms the importance of origin identification and PGI. Compared with the time-consuming HPLC method, Vis-NIR spectroscopy could predict the origin of *G. elata* and the contents of eight physiologically active ingredients in a single scan within seconds, thereby evaluating the quality of *G. elata*. This method is rapid, simple, non-polluting, and has lower instrument costs compared with HPLC instruments, demonstrating the necessity of adopting Vis-NIR spectroscopy for the rapid quality assessment of *G. elata*. However, to achieve the rapid quality inspection of *G. elata* in different scenarios, it is necessary to develop corresponding portable devices for Vis-NIR spectroscopy.

The Vis-NIR models that were established using the 1D-CNN nonlinear method outperformed other tested conventional models, indicating that there were more inherent nonlinear correlations between spectral data and origin labels or content. In particular, the 1D-CNN method based on deep learning had advantages, such as automatic feature extraction, hierarchical feature learning, parameter sharing and local perception, data augmentation, and generalization ability. If applied to portable devices for Vis-NIR spectroscopy, it could better handle local features in the data, make the model more adaptable, simplify the model construction process, and further the generalization ability of the model.

The phenolics in *G. elata* have neuroprotective, anti-inflammatory, and antioxidant effects. Therefore, besides being used as a health supplement, *G. elata* is also applied as a drug in clinical applications [46]. For instance, Tianma injection was applied to cure a patient who had vertebrobasilar insufficiency [47]. Tianmasu injection was applied to cure a patient who had dizziness [48]. *G. elata* as a vegetable medicine is more and more welcome in some countries. In future research, more *G. elata* samples will be collected from different origins to expand the application range and improve the reliability of the proposed models. Additionally, in order to produce various high-quality end products, including food supplements and medications, economical and portable Vis-NIR equipment combined with the advantages of a 1D-CNN will be developed, which will meet the demand for rapid quality inspection of *G. elata* products for industry use.

## 5. Conclusions

In this study, Vis-NIR spectroscopy combined with chemometric methods (PLS-DA, KNN, SVM, 1D-CNN) was applied to correctly and rapidly identify the geographical origin of *G. elata* and predict the contents of its physiologically active ingredients. First, identification models using the four algorithms were applied to differentiate between origins. The 1D-CNN had a good performance that could correctly identify all the origins, with the F1 score being 1.0000. Second, Vis-NIR spectroscopy was applied to quantify the contents of eight physiologically active ingredients in *G. elata* from different origins. Four quantitative models were constructed, and their prediction performances were compared. The 1D-CNN performed better than the other three models. In the prediction sets, the *RMSEP* values for GA, HA, PE, PB, PC, PA, GA + HA, and total were 0.2881, 0.0871, 0.3387, 0.2485, 0.0761, 0.7027, 0.3664, and 1.2965, respectively. The $R_{p}^{2}$ values were 0.9278, 0.9321, 0.9433, 0.9094, 0.9454, 0.9282, 0.9173, and 0.9323, respectively. These results confirm the potential of combining Vis-NIR technology with a 1D-CNN for *G. elata* quality evaluation. In future research, more *G. elata* samples from different production regions and harvesting times will be collected to enhance the reliability and applicability of the models. Additionally, taking advantage of a 1D-CNN, more affordable and portable Vis-NIR devices will be developed for the quality assessment of *G. elata* in the market.

## Figures and Tables

**Table 1:** Geographical information of the *G. elata* samples.

City	Province	Number of Batches	Region (City)	Region (Province)	Number of Batches
Dejiang (DJ)	Guizhou	25	Zhaotong (ZT)	Yunnan	5
Dafang (DAF)	Guizhou	40	Yichang (YC)	Hubei	20
Leishan (LS)	Guizhou	40	Wufeng (WF)	Hubei	5
Pu’an (PUA)	Guizhou	25	Lueyang (LY)	Shaanxi	30
Liping (LP)	Guizhou	25	Danfeng (DF)	Shaanxi	20
Lijiang (LJ)	Yunnan	5			

**Table 2:** Network structural parameters of 1D-CNN.

Network Layer	*G. elata*		
	Input Shape	Output Shape	Hyperparameters
Gaussian noise	(None, 1030)	(None, 1030)	t = 0.05
Reshape	(None, 1030)	(None, 1030, 1)	
1D convolution	(None, 1030, 1)	(None, 999, 8)	K = 8, s = 32, a = “ReLU”
1D convolution	(None, 999, 8)	(None, 968, 16)	K = 16, s = 32, a = “ReLU”
Dropout	(None, 968, 16)	(None, 968, 16)	r = 0.5
Flatten	(None, 968, 16)	(None, 15,488)	
Dense	(None, 15,488)	(None, 128)	d = 128, a = “ReLU”
Output	(None, 128)	(None, 11)	d =11, a = “Softmax”

t: standard deviation of the generated Gaussian noise, k: number of the convolution kernels, s: size of each kernel, a: activation function, r: dropout rate of the input units, d: output spatial dimensions, ReLU: rectified linear unit.

**Table 3:** Classification statistics of the *G. elata* samples.

Samples	SPXY Divided the Sample Set	Number of Samples	Min (mg/g)	Max (mg/g)	Mean (mg/g)	Std (mg/g)
Gastrodin (GA)	Training set	180	0.4172	8.7501	2.0922	1.6732
	Testing set	60	0.4172	6.1368	1.6160	1.0809
p-Hydroxybenzyl alcohol (HA)	Training set	180	0.2654	2.1445	0.8901	0.4285
	Testing set	60	0.2654	1.6148	0.7957	0.3369
Parishin E (PE)	Training set	180	1.3070	7.0529	3.3809	1.3630
	Testing set	60	1.3070	6.2551	3.5147	1.4350
Parishin B (PB)	Training set	180	0.9511	5.1121	2.9258	0.9635
	Testing set	60	0.9511	4.9068	2.6958	0.8327
Parishin C (PC)	Training set	180	0.2227	1.9953	0.7953	0.3920
	Testing set	60	0.2227	1.8459	0.7049	0.3285
Parishin A (PA)	Training set	180	0.9179	15.9855	6.4430	3.3290
	Testing set	60	0.9179	12.1205	5.3990	2.6437
GA + HA	Training set	180	1.0952	10.4906	2.9512	1.9493
	Testing set	60	1.0952	7.4369	2.4284	1.2823
Total	Training set	180	5.2818	33.2749	16.4603	6.3342
	Testing set	60	5.2818	29.5875	14.5303	5.0405

GA + HA: the sum of gastrodin and p-hydroxybenzyl alcohol; total: the sum of gastrodin, p-hydroxybenzyl alcohol, parishin E, parishin B, parishin C, and parishin A; SPXY: sample set portioning based on joint x–y distance; Min: minimum value; Max: maximum value; Mean: mean value; Std: standard deviation.

**Table 4:** Bioactive component contents of *G. elata* samples from different origins (mg/g).

Region of Origin	Gastrodin (GA)	p-Hydroxybenzyl Alcohol (HA)	Parishin E (PE)	Parishin B (PB)	Parishin C (PC)	Parishin A (PA)	GA + HA	Total
Dafang (DaF)	1.5117	0.3898	3.7303	2.2392	0.6245	3.5280	1.9015	12.0235
Pu’an (PA)	3.5800	1.1643	2.9929	3.9372	1.1369	9.7448	4.7443	22.5561
Yingchang (YC)	1.0361	0.9661	3.8818	2.4010	0.4814	4.7407	2.0021	13.5071
Wufeng (WF)	1.2197	0.8885	4.2984	2.7102	0.5647	6.0205	2.1082	15.7020
Lijiang (LJ)	2.0826	1.2167	2.7562	3.0411	0.6751	3.3120	3.2993	13.0837
Zhaotong (ZT)	1.3222	1.1378	4.9121	2.5771	0.5723	2.6593	2.4600	13.1809
Lueyang (LY)	1.5378	0.6945	1.9492	2.6026	0.7956	6.7290	2.2323	14.3087
Liping (LP)	1.1844	0.5356	5.8001	2.6807	0.4926	5.8463	1.7200	16.5397
Leishan (LS)	1.5864	1.2674	3.7008	3.1863	0.7102	6.3293	2.8538	16.7804
Danfeng (DF)	1.2382	0.5367	2.7307	2.0601	0.5670	3.4004	1.7749	10.5331
Dejiang (DJ)	4.7040	1.1842	2.6924	3.9674	1.5170	10.3782	5.8882	24.4433
Mean	1.9094	0.9074	3.5859	2.8548	0.7398	5.6989	2.8168	15.6962
CV%	61.06	35.09	31.23	22.05	42.74	44.98	47.8204	27.4375

Mean: mean value; CV: coefficient of variation; GA + HA: the sum of gastrodin and p-hydroxybenzyl alcohol; total: the sum of gastrodin, p-hydroxybenzyl alcohol, parishin E, parishin B, parishin C, and parishin A.

**Table 5:** Comparison of *G. elata* origin discrimination models based on different modeling methods.

Data Augmentation	Pre-Processing	Modeling Method	Acc_Train	Acc_Test	Precision	Recall Rate	F1 Score
No	Raw	PLS-DA	0.7833	0.8167	0.7995	0.8167	0.8031
		KNN	0.7555	0.8167	0.8417	0.8167	0.8064
		SVM	0.4167	0.4500	0.4366	0.4500	0.3750
		1D-CNN	0.2611	0.2500	0.1131	0.2500	0.1392
	Normalization	PLS-DA	0.8889	0.9167	0.9102	0.9167	0.9085
		KNN	1.0000	0.5167	0.5060	0.5167	0.5062
		SVM	0.8278	0.6500	0.7211	0.6500	0.6579
		1D-CNN	1.0000	0.9167	0.9142	0.9167	0.9067
	SD + Normalization	PLS-DA	0.9429	0.9556	0.9500	0.9440	0.9500
		KNN	0.9944	0.9667	0.9542	0.9667	0.9583
		SVM	0.9611	0.9833	0.9847	0.9833	0.9828
		1D-CNN	1.0000	1.0000	1.0000	1.0000	1.0000
Yes	Raw	PLS-DA	0.7125	0.7167	0.6845	0.7167	0.6706
		KNN	0.8292	0.7167	0.8539	0.7167	0.7170
		SVM	0.3833	0.4500	0.2933	0.4500	0.3241
		1D-CNN	0.3951	0.4667	0.3495	0.4667	0.3641
	Normalization	PLS-DA	0.9667	0.9167	0.9117	0.9167	0.9096
		KNN	1.0000	0.7167	0.7409	0.7167	0.7121
		SVM	0.8625	0.8833	0.9012	0.8833	0.8718
		1D-CNN	1.0000	0.9833	0.9847	0.9833	0.9833
	SD + Normalization	PLS-DA	0.9764	0.9833	0.9701	0.9833	0.9759
		KNN	1.0000	0.9833	0.9861	0.9833	0.9829
		SVM	1.0000	0.9833	0.9917	0.9833	0.9849
		**1D-CNN**	**1.0000**	**1.0000**	**1.0000**	**1.0000**	**1.0000**

Bolded font indicates the optimal modeling results; SD: second-order derivative; Acc_train: training set accuracy; Acc_test: testing set accuracy.

**Table 6:** Results of the calibration models for predicting the contents of bioactive components in *G. elata* based on different algorithms.

Number	Components	Spectral Pre-Processing	Modeling Method	$R_{v}^{2}$	MRECV	RMSECV	$R_{p}^{2}$	MREP	RMSEP
1	Gastrodin (GA)	SD	PLSR	0.9770	0.1277	0.2530	0.6794	0.3868	0.6069
			KNN	0.8835	0.1968	0.5696	0.8995	0.1964	0.3398
			SVR	0.9974	0.0549	0.0851	0.8869	0.2360	0.3604
			1D-CNN	0.9867	0.0707	0.1924	0.8913	0.1920	0.3535
		SD + augmentation	PLSR	0.9749	0.1323	0.2642	0.6775	0.3864	0.6086
			KNN	0.9247	0.1722	0.4577	0.9202	0.1714	0.3027
			SVR	0.9985	0.0385	0.0644	0.8863	0.2243	0.3615
			**1D-CNN**	**0.9974**	**0.0328**	**0.0843**	**0.9278**	**0.1396**	**0.2881**
2	p-Hydroxybenzyl alcohol (HA)	SD	PLSR	0.9179	0.1463	0.1224	0.6796	0.2459	0.1918
			KNN	0.941	0.0952	0.1038	0.89	0.1207	0.1108
			SVR	0.9644	0.1094	0.0806	0.8749	0.1596	0.1182
			1D-CNN	0.9744	0.072	0.0684	0.919	0.1184	0.0951
		SD + augmentation	PLSR	0.9346	0.1270	0.1093	0.6715	0.2602	0.1915
			KNN	0.8761	0.1143	0.1505	0.9274	0.0891	0.09
			SVR	0.9746	0.0926	0.0681	0.8972	0.1412	0.1071
			**1D-CNN**	**0.9976**	**0.0201**	**0.021**	**0.9321**	**0.0884**	**0.0871**
3	Parishin E (PE)	SD	PLSR	0.856	0.1468	0.5158	0.8862	0.1196	0.4801
			KNN	0.8810	0.1153	0.4689	0.8858	0.1143	0.4810
			SVR	0.8499	0.1183	0.5267	0.8800	0.1228	0.4930
			1D-CNN	0.9971	0.018	0.0735	0.8963	0.1166	0.4583
		SD + augmentation	PLSR	0.8522	0.1479	0.5226	0.885	0.1197	0.4825
			KNN	0.9381	0.0763	0.3382	0.9241	0.0902	0.3919
			SVR	0.9972	0.0218	0.0725	0.9405	0.0831	0.3471
			**1D-CNN**	**0.9978**	**0.0164**	**0.0634**	**0.9433**	**0.0839**	**0.3387**
4	Parishin B (PB)	SD	PLSR	0.801	0.1409	0.4286	0.6413	0.1704	0.4945
			KNN	0.9166	0.0867	0.2775	0.8345	0.1139	0.3359
			SVR	0.9907	0.0328	0.0925	0.9066	0.0849	0.2523
			1D-CNN	0.9788	0.0388	0.1396	0.8978	0.0812	0.2639
		SD + augmentation	PLSR	0.7998	0.141	0.4299	0.6408	0.1710	0.4949
			KNN	0.826	0.1021	0.4008	0.8623	0.0776	0.3064
			SVR	0.9951	0.0228	0.0670	0.9243	0.0752	0.2271
			**1D-CNN**	**0.9969**	**0.0151**	**0.0528**	**0.9094**	**0.0788**	**0.2485**
5	Parishin C (PC)	SD	PLSR	0.8844	0.164	0.1329	0.7178	0.2076	0.1731
			KNN	0.9589	0.0926	0.0792	0.9087	0.1216	0.0984
			SVR	0.9572	0.1219	0.0808	0.8514	0.1901	0.1256
			1D-CNN	0.985	0.0601	0.0478	0.9304	0.1176	0.0859
		SD + augmentation	PLSR	0.8709	0.1762	0.1405	0.7201	0.2141	0.1723
			KNN	0.9119	0.1031	0.116	0.9373	0.0885	0.0816
			SVR	0.9691	0.1001	0.0688	0.8691	0.1712	0.1179
			**1D-CNN**	**0.9941**	**0.0335**	**0.0299**	**0.9454**	**0.0887**	**0.0761**
6	Parishin A (PA)	SD	PLSR	0.8934	0.2027	1.0839	0.6216	0.3181	1.6127
			KNN	0.9359	0.1334	0.8402	0.8439	0.2031	1.0358
			SVR	0.9904	0.0494	0.3244	0.8955	0.1967	0.8474
			1D-CNN	0.9950	0.0379	0.2358	0.9448	0.1215	0.6159
		SD + augmentation	PLSR	0.8985	0.1922	1.0577	0.6228	0.3160	1.6101
			KNN	0.9089	0.1362	1.0018	0.9269	0.1079	0.7086
			SVR	0.9990	0.0204	0.1032	0.9078	0.1690	0.7959
			**1D-CNN**	**0.9985**	**0.0247**	**0.1274**	**0.9282**	**0.1329**	**0.7027**
7	GA + HA	SD	PLSR	0.9741	0.1089	0.3129	0.6699	0.2989	0.7321
			KNN	0.9191	0.1465	0.5530	0.8946	0.1486	0.4136
			SVR	0.9976	0.0351	0.0942	0.9037	0.1618	0.3954
			1D-CNN	0.9970	0.0298	0.1047	0.9015	0.1216	0.3999
		SD + augmentation	PLSR	0.9704	0.1132	0.3344	0.6718	0.2994	0.7300
			KNN	0.9483	0.1202	0.4420	0.9141	0.1331	0.3733
			SVR	0.9988	0.0251	0.0667	0.8990	0.1584	0.4048
			**1D-CNN**	**0.9976**	**0.0254**	**0.0958**	**0.9173**	**0.1006**	**0.3664**
8	Total	SD	PLSR	0.8928	0.1257	2.0679	0.6419	0.1830	2.9827
			KNN	0.9388	0.0872	1.5628	0.8649	0.1156	1.8318
			SVR	0.9370	0.0863	1.5851	0.8485	0.1314	1.9400
			1D-CNN	0.9991	0.0097	0.1926	0.9136	0.0861	1.4653
		SD + augmentation	PLSR	0.8897	0.1272	2.0975	0.6435	0.1826	2.9757
			KNN	0.9789	0.0375	0.9175	0.9261	0.0775	1.3547
			SVR	0.9983	0.0137	0.2580	0.9301	0.0864	1.3180
			**1D-CNN**	**0.9962**	**0.0202**	**0.3912**	**0.9323**	**0.0794**	**1.2965**

GA + HA: the sum of gastrodin and p-hydroxybenzyl alcohol; total: the sum of gastrodin, p-hydroxybenzyl alcohol, parishin E, parishin B, parishin C, and parishin A; bolded font indicates the optimal modelling result; $R_{v\;}^{2}$ is the coefficient of determination of the training set; MRECV: mean relative error for cross-validation; RMSECV: root-mean-standard error for cross-validation; $R_{p}^{2}$ is the coefficient of determination of the testing set; MREP: mean relative error for prediction; RMSEP: root mean standard error for prediction.

## Data Availability

The data in this study are available from the following sources: the corresponding authors. These data are not publicly available due to the requirement to fund research projects.
